# The musculotendinous interface: insights into development, injury, and recovery for military medical applications

**DOI:** 10.3389/fphys.2025.1555199

**Published:** 2025-05-06

**Authors:** Alex T. Adams, Zachary G. Davis, Kyle F. Browder, Christopher L. Dearth, Stephen M. Goldman

**Affiliations:** ^1^ Extremity Trauma and Amputation Center of Excellence, Defense Health Agency, Falls Church, VA, United States; ^2^ Department of Surgery, Uniformed Services University of the Health Sciences, Bethesda, MD, United States

**Keywords:** cumulative trauma disorders, military personnel, tendon injuries, wound healing, biomechanics

## Abstract

Musculoskeletal injuries (MSKIs) are a major cause of morbidity and lost duty time for military service members, impacting overall military readiness, with overuse injuries being particularly prevalent among them. Musculotendinous injuries, affecting the musculotendinous unit, are especially problematic due to their long recovery times and limited treatment options. To better understand these injuries, this review delves into the developmental, homeostatic, and structural biology of musculotendinous units, with a focus on the musculotendinous junction (MTJ). Additionally, it explores the biomechanical model of the musculotendinous unit and the complexities of endogenous repair processes for muscle, tendon, and MTJ injuries. Based on these insights, the review discusses promising therapeutic approaches for treating these injuries, such as anabolic agents, metabolic reprogramming, scaffold or cell-based therapies, and physical therapy. These emerging therapies offer potential avenues for accelerating endogenous healing, reducing recovery time, and improving long-term outcomes for musculotendinous injuries. Ultimately, further research in this area could significantly enhance military readiness by mitigating the impact of MSKIs on service members.

## Introduction

Musculoskeletal injuries (MSKIs) can occur at any point in the career of a U.S. Service member and are a persistent issue within the military health system (Reis et al., 2007; Darakjy et al., 2006; Patel et al., 2017). The impact of MSKIs on military readiness is substantial as they are the leading cause of outpatient encounters in the military health system (Molloy et al., 2020a; Grimm et al., 2019; Molloy et al., 2020b; Lovalekar et al., 2023) they account for nearly 60% of limited duty days (Molloy et al., 2020a; Sammito et al., 2021) and up to 50% of disease and non-battle injury casualties (Sammito et al., 2021). As such, the Department of Defense is actively working to develop more effective prevention and treatment strategies to mitigate their effects with the goal of limiting the impact on military readiness, and reducing injury recurrence.

Although combat-related MSKIs greatly impact the United States military, non-combat-related MSKIs pose a more pervasive threat to troop readiness (Molloy et al., 2020a; Grimm et al., 2019; Molloy et al., 2020b; Lovalekar et al., 2023). The majority of MSKIs, approximately 70%, are overuse injuries that occur during training, rather than in combat (Molloy et al., 2020a; Molloy et al., 2020b). If left unaddressed, these overuse injuries can become exacerbated, ultimately impeding service members’ ability to perform their duties effectively. A study of 930 service members revealed that 61% experienced significant pain during training, primarily in the foot, ankle, and upper leg. Notably, 11% of those who experienced pain were unable to complete their training due to the severity of their symptoms (Keijsers et al., 2022). Service members in non-combat units are disproportionately affected, experiencing higher rates of MSKIs, limited duty days, and chronic MSKIs, compared to those in combat arms units (Molloy et al., 2020a; Molloy et al., 2020b; Teyhen et al., 2018). Furthermore, prior injury was shown to be a significant predictor of MSKIs in a recent meta-analysis, emphasizing the longstanding impact of MSKI on military readiness (Rhon et al., 2022).

A specific category of MSKIs, including musculotendinous conditions such as Achilles tendinitis, plantar fasciitis, bursitis, patellofemoral syndrome, as well as sprains, strains, and ruptures, is of particular concern due to the prolonged recovery periods associated with these conditions. These soft tissue overuse injuries represent a substantial burden on the military health system, accounting for 41.7% of hospitalizations and 86.1% of outpatient visits among all musculoskeletal conditions. Moreover, they are estimated to cause approximately 3.8 million limited-duty days annually, significantly impacting military readiness (Molloy et al., 2020a; Jones et al., 2010). Furthermore, current treatment options for these conditions are limited and often require extended periods of recovery, taking several months to a full year (Silbernagel et al., 2020). A deeper understanding of the mechanisms behind these musculotendinous injuries may inform new treatments that reduce recovery time and pain for service members. This will decrease lost duty days and medical costs, ultimately improving military readiness.

### Developmental biology of musculotendinous units

The formation of the musculoskeletal system is a complex, multistep process that involves intricate actions to correctly assemble muscles, tendons, and bones. These actions necessitate constant communication between different cell types to organize and construct the unique tissues (He et al., 2022; Huang, 2017). During development, tendons attach contractile tissue to bones, enabling efficient movement (Subramanian and Schilling, 2015; Schweitzer et al., 2010). The formation of tendons occurs in three stages: induction, organization, and differentiation of progenitor cells (Schweitzer et al., 2010).

The somite, an axial structure found in embryos, is divided into the ventromedial sclerotome and the dermomyotome (Brent et al., 2003; Tani et al., 2020). The sclerotome gives rise to bones, and the dermomyotome gives rise to both the dermis and the myotome, the muscle component of the musculoskeletal system (Tani et al., 2020). Tendons in the trunk of the body are formed in the somite region of vertebrates (He et al., 2022; Schweitzer et al., 2010; Brent et al., 2003). Induction of tendon progenitor cells occurs between a neighboring myotome and sclerotome, producing the syndetome (Schweitzer et al., 2010). The syndetome contains tenocyte progenitor cells (TPCs), which result from fibroblast growth factor (FGF) released from the myotome (Subramanian and Schilling, 2015; Brent et al., 2003). These TPCs express the bHLH transcription factor scleraxis (Scx), a marker of tendon cells from early embryonic stages and throughout development (He et al., 2022; Brent et al., 2003; Andarawis-Puri et al., 2015; Shukunami et al., 2018; Shukunami et al., 2006). Although the same major signaling molecules curate tendon development throughout the body, slight differences in tissue interactions and cell dynamics exist in the primary sections of the body during induction (He et al., 2022).

**FIGURE 1:**
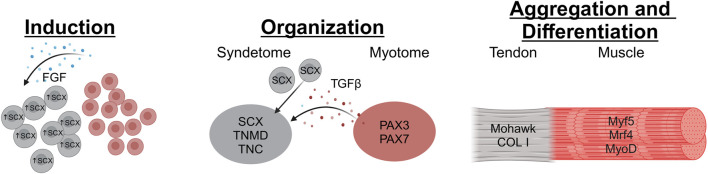
The stages of tendon development begin with Induction, where FGF (possibly secreted by muscle progenitor cells in the myotome) stimulates Scx expression in tendon progenitor cells. Then during the Organization stage, the tendon progenitors organize into loose collections of cells situated between the progenitor muscle and bone tissues. This is contingent on TGFβ signaling from the Myotome which recruits tendon progenitors to the location. Finally, in the Aggregation and Differentiation stage the tendon progenitors condense and start laying down matrix to create the tendon structure (Image created at https://BioRender.com).

Induction of tendons (Figure 1) in the limbs occurs in an early limb bud arising from the lateral plate mesoderm (Huang, 2017; Schweitzer et al., 2010). Scx expression during limb tendon development is not location-specific in relation to skeletal or muscular progenitors. Instead, progenitors for all components of the musculoskeletal system (bone, muscle, and tendon) are intermixed within the early limb bud before organization (Huang, 2017; Edom-Vovard and Duprez, 2004). Early limb tendon induction is independent of signals from nearby muscles; however, these signals are crucial for later differentiation (Schweitzer et al., 2001; Kieny and Chevallier, 1979; Kardon, 1998). Studies using mouse and chick models have revealed key aspects of early tendon formation. Notably, these models demonstrate that the initial induction of tendon progenitor cells, marked by the presence of Scleraxis (Scx)-expressing cells, can occur even in the absence of muscle tissue (Subramanian and Schilling, 2015; Kieny and Chevallier, 1979; Kardon, 1998; Gumucio et al., 2020). In the nascent limb bud, the ectoderm has been identified as the essential tissue source for signals driving these very early stages of tendon induction (He et al., 2022; Schweitzer et al., 2001). As the limb develops, this tendon induction process follows a characteristic pattern, progressing from the proximal (closer to the body) to the distal (further from the body) regions (Edom-Vovard and Duprez, 2004). FGF signaling is essential throughout limb tendon development (Tani et al., 2020; Bessho and Kageyama, 2003), mediating a positive feedback loop of paracrine signaling between mesenchymal and epithelial tissues. This interplay is fundamental not only for tendon development but also for patterning the overall limb bud, stimulating its outgrowth and morphogenesis, and maintaining the integrity of the early limb structure (Teven et al., 2014). These findings show high translatability to humans due to deeply conserved developmental mechanisms. Key signaling pathways (like FGF) and transcription factors (like Scx) operate similarly across species, and the essential dialogue between ectoderm and mesenchyme, driving proximal-to-distal limb formation, is conserved (Pownall and Isaacs, 2010). Evidence suggests initial tendon specification occurs independently of muscle in humans, mirroring model organisms, and the critical role of pathways like FGF is confirmed by human genetic disorders causing limb defects (Wenger et al., 2020).

The last step of tendon progenitor cell manipulation and recruitment is differentiation, which completes the musculotendinous junction and tendon-bone assembly in vertebrates. Tendon progenitor cells condense into distinct structures, mainly composed of type I collagen, and integrate with muscle and pre-bone cartilage condensation to form anchor points (Schweitzer et al., 2001; Pryce et al., 2009). Tendons in the limbs vary in length, thickness, and strength. The differentiation that occurs depends on the nearby muscle location and function as well as the presence of Scx, due to the variety in structural integrity required for tendons in the limbs (He et al., 2022). For example, Pax3 knockout mice, which lack muscle, show the initial stages of tendon development, but the tendons fail to elongate due to the absence of muscle (Huang et al., 2015; Theodossiou and Schiele, 2019). As a result, tendons in the distal part of the limbs (lower presence of nearby muscle) are structurally different and often less resistant to high forces than those in the proximal part of the limbs (Kardon, 1998).

Skeletal muscle development in vertebrates shares similarities with tendon development in its complexity and multi-stage nature. Both originate from specific progenitor cells that undergo induction and differentiation processes. In the case of skeletal muscle, the journey begins in the somite, specifically within the dermomyotome compartment. Cells destined to become muscle receive signals that prompt their relocation to the myotome (Buckingham, 2001; Buckingham et al., 2003). Once within the myotome, and particularly in the context of limb development, these progenitor cells activate a cascade of gene expression. Initially, they express Pax3 and Pax7, transcription factors that are indicative of their myogenic potential. Subsequently, they upregulate the expression of Myf5, Myog, and Myod. These key regulatory factors drive the further differentiation of the progenitor cells into myoblasts, which are the precursors of muscle fibers. The myoblasts then undergo a series of morphological and functional changes, including cell fusion and the assembly of contractile proteins, ultimately leading to the formation of mature myofibers (Endo, 2015; Sato, 2020).

The development of both tissues in the musculoskeletal system occurs simultaneously as the vertebrate grows (Gaut and Duprez, 2016). The complex process of tendon and muscle development becomes far more dependent on one another at their connection point, the musculotendinous junction (MTJ) (Gaffney et al., 2023; Gaffney et al., 2022).

### Homeostatic and structural biology of musculotendinous units

After the complete development of the extremity tendons and muscles, the regulation of healthy musculotendinous units depends on their specific structure, surrounding bones and muscles, and the mechanical forces applied to them (Gaffney et al., 2023; Gaffney et al., 2022). In the most distal section of limbs, tendons are categorized into two groups: extensor and flexor tendons (Benjamin et al., 2008). These two different tendons help transmit muscular contractions to the skeleton allowing the body to move at different speeds and with different amounts of force (Benjamin et al., 2008). For tendons and muscles in the extremities to function properly, they must be maintained effectively and efficiently.

Tendons largely consist of type 1 collagen (65%–80% by dry mass), tenocytes, and proteoglycans in the extracellular matrix (ECM) (Kannus, 2000; Silver et al., 2003). Proteoglycans function as the viscoelastic component of tendon ECM contributing little to the tensile strength (Silver et al., 2003; Puxkandl et al., 2002; Sharma and Maffulli, 2005). Collagen, on the other hand, is responsible for resisting tensile forces the tendon is subjected to while providing some flexibility for range-of-motion. Tendons have a hierarchical and helical structure resembling a man-made rope, providing torsional strength and flexibility (Silver et al., 2003; Bozec et al., 2007; Liu et al., 1995). Tenocytes, primary tendon fibroblasts, reside along the collagen fibrils and run longitudinally to the long axis of the fibrils secreting extracellular matrix (Thorpe and Screen, 2016). The tenocyte proliferation and collagen production are regulated by Tenodulin, a molecule induced by Scx (Shukunami et al., 2018; Gaut and Duprez, 2016; Docheva et al., 2005).

Mature tendon cells are responsible for intracellular communication and regulation of the tendon (Gaut and Duprez, 2016). Mechanical stress leads to a strong response from these cells and is essential in maintaining strength within the tendon (Silver et al., 2003). Studies have shown that during a single period of acute exercise, collagen synthesis in the patellar tendon increases 100% and is still evident 3 days post-workout (Miller et al., 2005). Tenascin-C is expressed when tenocytes experience mechanical load and regulates cell migration and proliferation through pro-inflammatory cytokines (Chiquet-Ehrismann and Tucker, 2004; Midwood and Orend, 2009). During periods of tension and stress, tendons will restructure during repair to adapt to the increased loading environment (Liu et al., 1995). In these moments, cell signaling occurs via gap junctions. Through these channels, the tenocytes interact constantly with the proteoglycans and other cells in the ECM to adapt collagen production leading to the restructuring of the tendon (Zabrzyński et al., 2018). Importantly, cell proliferation is induced by short periods of tensile stress, but inhibited by long periods of mechanical loading (Barkhausen et al., 2003). Persistent mechanical loading over time can lead to tendinopathy or ruptured tendon tissue (Sharma and Maffulli, 2005; Zabrzyński et al., 2018).

Skeletal muscle is responsible for converting chemical energy into mechanical energy producing force and power to move the body (Frontera and Ochala, 2015). Skeletal muscle is an incredibly dynamic tissue and is composed of hierarchically organized fascicles (Mukund and Subramaniam, 2020). Fascicles are comprised of bundled muscle fibers which are comprised of myofibrils (Mukund and Subramaniam, 2020). Myofibrils are made up of myofilaments which are arranged into sarcomeres, the contractile portion of the muscle (Frontera and Ochala, 2015). Each muscle fiber contains thousands of myofibrils containing billions of myofilaments (Frontera and Ochala, 2015). This organized system works together to create strong contractions allowing the body to move. For the conversion of chemical energy into mechanical energy, many proteins and intracellular signals are involved. The primary proteins involved are actin and myosin, which make up the myofilaments, troponin and tropomyosin, which allow for the sliding of myofilaments creating a contraction, and titin and nebulin, which contribute to the structural stability of the sarcomere (Frontera and Ochala, 2015; Ottenheijm and Granzier, 2010; Monroy et al., 2012). For contraction to take place, calcium must be released into the sarcoplasm allowing the overlap of myofilaments leading to them sliding past one another shortening the muscle fiber (Huxley and Niedergerke, 1954). This signaling relies on many things including the nervous system, muscle size, the number of myofilaments available, the space between filaments, and the quality of the intracellular signaling (Frontera and Ochala, 2015).

As for the homeostasis biology of muscle, a great example can be seen when analyzing the impact of exercise on skeletal muscle. Training in any capacity (endurance or strength) greatly alters the structure and metabolic activity of the muscle and its components (Lamon et al., 2014). Similar to tendons, muscles will adapt to unique situations when provided with a mechanical stimulus. During endurance training, capillary supply to muscles increases, mitochondria presence increases and degraded mitochondria are removed with greater efficiency, and glycogen stores are increased to handle the load (Yan et al., 2012; Hearris et al., 2018; Murray and Rosenbloom, 2018). There is also an increase in muscle fiber efficiency with a mature sarcoplasmic reticulum and reduced presence of calcium-interacting proteins (Green et al., 2003). On the other hand, strength training improves the ability to generate power and force through muscle hypertrophy-increased size of individual muscle fibers due to additional myofilaments, myofibrils, and sarcomeres (Frontera and Ochala, 2015). Hypertrophy has been linked to the increase of IGF-1 and the upregulation of the Myostatin pathway (Frontera and Ochala, 2015). These structural changes to both tendon and muscle rely on intracellular communication and cell-matrix interactions in response to different environmental cues (Kjær, 2004).

### Cross-talk during the development of the musculotendinous junction

Throughout development, the belly of the muscle and the center of the tendons develop as previously stated. However, at their connection point, the development of the MTJ displays significantly more cross-talk between the different tissues (Tidball, 1994; Charvet et al., 2012). During the late stages, the migration and maturation of tendons and muscle cells near the MTJ are dependent upon the other’s presence (Charvet et al., 2012). The presence of tendon precursors inhibits muscle cell migration while muscle cells induce tendon progenitor cells (Kardon, 1998). At the MTJ, mature muscle and tendon cells interact with one another through a rich extracellular matrix (ECM) forming the basement membrane (Kardon, 1998). The basement membrane is primarily composed of laminins, collagen IV, and thrombospondin (Adams and Lawler, 2004). Integrins, the primary ECM receptors in the basement membrane, are vital in MTJ development (Gaffney et al., 2022; Kjær, 2004). It has been shown that the absence of the *α*7 integrin leads to muscular dystrophy and is essential for muscle fiber attachment during MTJ development (Mayer et al., 1997). The basement membrane provides a strong support system for connecting tendon and muscle tissue.

Following basement membrane development, random contractions organize collagen fibers of the tendon, as well as thin and thick filaments of the muscle, into a parallel alignment (Kardon, 1998). As contractions continue, there is progressive formation and linking of muscle fibers and collagenous tendons (Charvet et al., 2012; Tidball and Lin, 1989). This indicates that there is not only a chemical cross-talk taking place during MTJ formation but a mechanical one as well.

### The unique properties and structure of the musculotendinous junction

**FIGURE 2:**
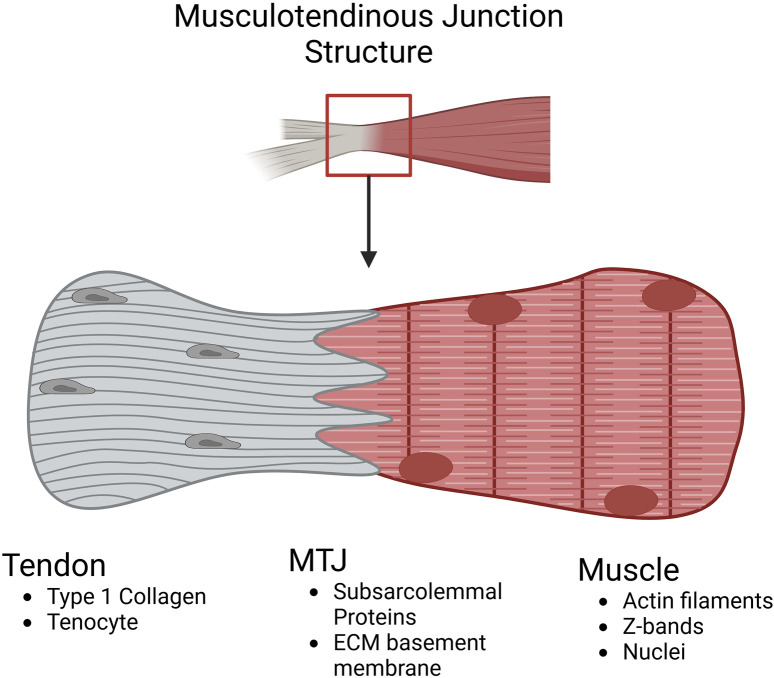
Schematic highlighting the compositional differences at the musculotendinous junction. In the tendon, the matrix is predominantly type I collagen and hypocellular with the main cell population being tenocytes (tendon fibroblasts). In the MTJ, integrins from the muscle link with the tendon ECM components throughout the dendritic projections intertwining muscle and tendon tissue. In the muscle, typical filament proteins like Actin and Myosin are present along with the myoblast cell population (Image created at https://BioRender.com).

The musculotendinous junction is the region where muscle and tendon interact and is the primary site of force transmission (Charvet et al., 2012). After attachment takes place, a dynamic and functional unit is produced that is responsible for the movement of the musculoskeletal system (Valdivia et al., 2017). Its structure is composed of both tendinous and muscular materials (Figure 2).

The muscle’s most distal and proximal portions contain myofilaments within the MTJ, which attach to the extracellular matrix proteins of the collagenous tendons (Charvet et al., 2012). The overlap occurs with finger-like protrusions from each component and varies based on the location and necessary strength of the tendon (Nakao, 1975). The muscular portion of the MTJ is actin filaments that are connected to the subsarcolemmal proteins that closely interact with the tendinous extracellular components (Kojima et al., 2008). One protein vital for forming the connection between the muscular filaments and the tendon ECM is paxillin which is seen as a marker for the MTJ due to its enriched presence (Gaffney et al., 2023; Jacob et al., 2017). Paxillin is a focal adhesion-associated adaptor protein that acts as a regulator of the ECM environment during development and as a check on cell spreading (Jacob et al., 2017; Schaller, 2001). Furthermore, the size and quantity of the components within the MTJ depend on slow or fast-twitch muscle fibers and the age of the muscle (Jakobsen et al., 2023). The finger-like protrusions are wider in slow twitch muscle compared to fast twitch muscle and after time, become shortened, decreasing the contact area between muscle and tendon resulting in an increase in injury (Jakobsen et al., 2023; Ciena et al., 2010; Trotter and Baca, 1987).

### Biomechanical model of the musculotendinous units

Planned and regulated by the nervous system, movement of the body takes place when muscles contract transmitting force through tendons which causes rotation of bones about a joint (Pandy and Barr, 2004). The speed, duration, and force generated by these muscular contractions depend on muscle size, muscle type, and level of activation dictated by the central nervous system.

Different models have been created to further understand and study the musculoskeletal system’s biomechanical properties. A widely accepted model of the musculotendinous system is the Hill model, which consists of a contractile element and two non-linear springs, one in parallel and one in series (Göktepe et al., 2014). The contractile unit in series with an elastic element represents the active portion of the muscle while the parallel elastic element with no contractile unit represents the passive portion of the muscle (Pandy and Barr, 2004). The muscular unit is then in series with the tendon modeling the whole system.

**FIGURE 3:**
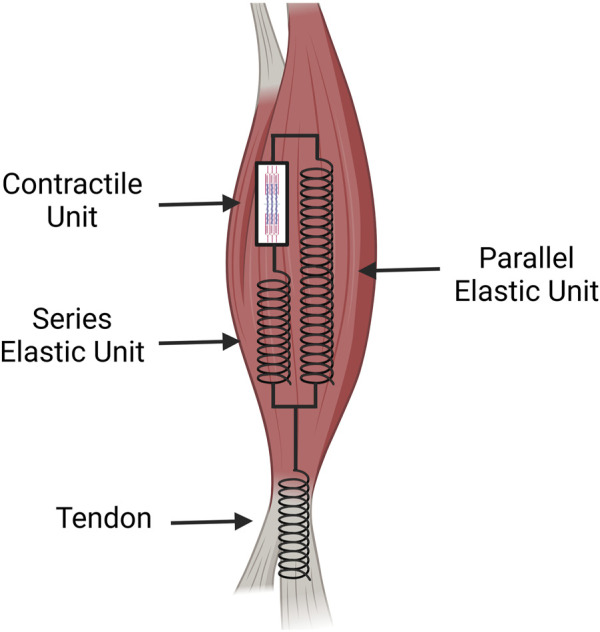
A spring and dashpot diagram of the tissue mechanics for the muscle and tendon. The muscle possesses the contractile unit which provides the initial force for movement. This force is transferred along a muscle’s ECM acting in series as an elastic unit that then transmits the force to the elastic component of the tendon. The parallel elastic unit of the muscle acts as the passive muscle stiffness which aids in recovery after contraction (Image created at https://BioRender.com).

The contractile unit in Figure 3 represents the sarcomeres within a muscle which produce force after receiving an electrical stimulus from the central nervous system (Pham and Puckett, 2024). The elastic units in series represent connective tissue and the ECM that is pulled in and compressed during muscular contraction within an active muscle. This creates a force that is then transferred through the tendon causing skeletal movement. The parallel elastic unit is also composed of connective tissue and the ECM but has a different function as the passive component of muscle (Gillies and Lieber, 2011). Although elastic, it is rigid and resists compression/stretching of the muscle (Göktepe et al., 2014; Gillies and Lieber, 2011). This model is used to quantitatively analyze the musculotendinous unit to better understand skeletal muscle physiology and biomechanics during movement. Using a single, nonlinear differential equation that contains musculotendon length, musculotendon force, musculotendon shortening velocity, and muscle activation, maximum musculotendon force can be calculated at specific instances (Pandy and Barr, 2004).

Although the simplicity of the Hill model and the equations derived from it provides a general framework for understanding the biomechanics, significant inter-individual variability exists in the components being modeled. For example, varying ratios of Type I (slow-twitch) and Type II (fast-twitch) fibers affect force-velocity relationships, fatigue resistance, and activation/deactivation times (Kissane et al., 2018; Edman et al., 2024) – all parameters within or relevant to Hill models. Moreover, tendon stiffness is known to vary with age (Stenroth et al., 1985), training history (Burgess et al., 2007; Reeves et al., 2003; Kubo et al., 1985), genetics (Passini et al., 2021; Mokone et al., 2006; Mokone et al., 2005), and sex (McMahon and Cook, 2024) which impacts force transmission, electromechanical delay, and the storage/release of elastic energy. As such, the development of subject-specific models which incorporate more of these parameters will be necessary for a more realistic model of the musculotendinous unit. Work has been done to expand upon the simplicity of the contractile unit in the Hill model by focusing on new data and findings regarding the myosin cross-bridge cycle (Seow, 2013). This inclusion helps the Hill model force-velocity relationship be a more well-rounded equation for analyzing small and large loads on the muscle. Over the last few decades, this model has been constantly refined to more accurately portray biological conditions and is used regularly to understand injuries and how to possibly prevent them.

Furthermore, due to the complexity and slight differences in muscle, bone, and tendon structures between individuals in different locations of the body, models have large limitations concerning how closely they can be interpreted. It is common practice to analyze single elements at a time within the body (i.e., femur, biceps, or cartilage of the knee) (Viceconti et al., 2006). Joint models have been created as a whole since these are primary locations of articulation and injury but have suffered from the difficulty in creating personalized models that can be used for analysis. Statically, each muscle relevant in whichever MTJ can be determined by MRI (Reid et al., 1994). However, due to the low elastic modulus of soft tissue, the muscles/tendons in moving people are always in a state of deformation (Sharafi et al., 2011). Also, in many models of the MTJ, muscles are isolated from surrounding structures simplifying the quantitative analysis. This leads to inaccuracies as it is known that during muscular contractions, muscles press up against other tissues which have not been included in the model successfully (Martins et al., 1998). Due to the MTJ’s viscoelastic properties, event-specific equations and analysis are required for current models (Hutter et al., 2000; Grega et al., 2020). These events include slow versus rapid movements and differing levels of loaded stress applied to the MTJ (Sharafi et al., 2011).

Although not perfect, constant efforts are being made to further the landscape of biomechanical modeling to further understand the mechanics of healthy and injured tissue or bone. In 2002, the Living Human Project was launched with the ambitious goals of developing a worldwide data repository of anatomical function data and simulation algorithms to create the first *in silico* model of the human musculoskeletal system (Viceconti et al., 2006). Efforts of this nature are ongoing and are necessary to create a patient-specific model that is reliable in helping devise treatment plans or to better understand unique injuries.

### Injuries to musculotendinous structures

The lower extremities contain the largest muscles and tendons within the body. Due to their size and force production, they are also one of the most common sites of injury within the body (Romero-Morales et al., 2024; Adirim and Cheng, 2003). While midsubstance tendon injuries are most prevalent overall, injuries to the musculotendinous junction (MTJ) region represent the next most common category. This vulnerability is particularly pronounced in military populations (Chan et al., 2019; Hoppes et al., 2013; Temple et al., 1998), whose injury risk often stems from the extreme demands, such as high exercise volume and intensity, placed upon their highly conditioned systems. This contrasts sharply with sedentary individuals, whose risk is typically linked to deconditioning and diminished movement control (Lurati, 2018). Counteracting these vulnerabilities with targeted exercise programs that emphasize strength, flexibility, and controlled movement can significantly reduce the risk of MTJ injuries in both populations (Stephenson et al., 2021; Lovalekar et al., 2021; Brushøj et al., 2008).

Given the unique physical stressors faced by military personnel, the types of musculotendinous injuries they experience generally fall into two major categories: traumatic injuries and overuse or cumulative trauma injuries (Vila Pouca et al., 2021). Traumatic injuries to the musculotendinous unit are frequently observed during basic training. In this setting, recruits often transition abruptly from relatively sedentary lifestyles to intense daily physical activity, a timeframe often insufficient for tissue adaptation, thereby increasing susceptibility to acute strains and tears (Bulzacchelli et al., 2014).

Beyond these acute traumatic events, overuse injuries represent another significant challenge within the military context. These injuries typically arise from sustained high-intensity training, as seen in young athletes, or consistent overuse patterns common in middle-aged and older adults (Tong et al., 2024). Illustrating this point within a military setting, a recent study identified overuse injuries to the musculotendinous unit as the most common musculoskeletal injury (MSKI) among U.S. Naval Special Forces Operators and students undergoing demanding SEAL Qualification Training and Crewman Qualification Training (Lovalekar et al., 2017). Implementing periodization strategies that alternate periods of high-intensity training with periods of active recovery can be essential in preventing overuse injuries.

While both traumatic and overuse injuries affect all Service members undergoing rigorous training and duties, susceptibility can differ between sexes. The existing literature indicates that female Service members tend to experience higher overall rates of musculotendinous injuries, particularly overuse injuries during basic training, compared to their male counterparts undergoing identical training regimens. However, while women may exhibit higher overall injury rates in many studies, men can experience different injury distributions. Depending on the specific military role and training demands, men might show higher rates of certain acute muscle strains related to explosive power requirements, although overuse injuries remain a frequent concern for them as well.

Regardless of the inciting cause—be it traumatic or overuse—or the sex of the individual, once an injury to the MTJ is sustained, patients often struggle to regain full function, frequently leading to long-term pain and discomfort (Baldino et al., 2016). The MTJ’s propensity for injury stems partly from its inherent structural properties, making it the second most common injury site (Garrett, 1996). Specifically, the distinct stiffnesses and cross-sectional areas of the muscle and tendon components mean that under traumatic loads leading to full tears, one component typically fails before the other (Shama et al., 2024). At this junction, the more compliant muscle tissue usually fails first under load, resulting in direct damage to the MTJ (VanDusen et al., 2015). Such MTJ tears are most commonly observed in the pectoralis major, gastrocnemius, and hamstring muscles (Kakwani et al., 2007; Woods et al., 2004), often occurring during intense muscle activity, particularly following a period of reduced muscle use. Furthermore, clinical observations highlight that traumatic MTJ injuries frequently happen during eccentric loading phases, where the muscle lengthens under tension (Tidball, 1991), adding another layer to understanding the mechanics of these debilitating injuries.

### Endogenous repair of skeletal muscle injuries

Injury to skeletal muscle can occur in many ways, often leading to decreased muscular force production and/or a change in myofibril structure (Tidball, 2011). Acute muscle injuries are the most common and can be due to lacerations, contusions, freezing, burning, exposure to toxins, or tearing when skeletal muscle bears a load past its maximum capacity (most frequently occurring during eccentric contractions) (Tidball, 2011). When a muscle is injured, an inflammatory response is produced which includes an influx of neutrophils and macrophages (Butterfield et al., 2006; Tu and Li, 2023). Neutrophils and macrophages invade the injured site and can reside for as long as 5 days removing cellular debris via phagocytosis (Tidball, 2005). Although neutrophils have been proven to cause further damage to surrounding muscular tissue, it is believed to be necessary for regeneration to take place (Butterfield et al., 2006). Within the injury site, macrophages are responsible for clearing debris and being rich sources of cytokines and growth factors (Tidball, 2005).

Recent studies have shown that muscle cells themselves play an important role in regeneration and muscle remodeling (Tidball, 2005). Muscle fibers and satellite cells can regulate the extravasation of inflammatory cells and modulate the role of inflammatory cells at the injury site (Tidball, 2005). Muscle-derived nitric oxide (NO) is an important muscle inflammation regulator and has been shown to decrease neutrophil-mediated lysis of muscle cells (Nguyen and Tidball, 2003a). NO serves as a protecting agent of muscle and changes in NO synthase (NOS) have been shown to strongly influence the immune response at the injury site in the muscle (Balon and Nadler, 1994). NOS expression is positively regulated by muscle contractions and exercise, leading to healthier muscles by preventing further breakdown of muscle fibers by inflammatory cells (Tidball et al., 1998). An experiment by Nguyen et al. showed that mice expressing a muscle-specific NOS that received muscle loading post-injury had significantly lower neutrophil invasion compared to mice that did not receive muscle loading (Nguyen and Tidball, 2003b). Although macrophage invasion was not affected by loading, the unloaded muscle group was observed to have lesions in the muscle membrane while the loaded muscle group did not. This supports the idea that NO regulates neutrophil invasion in the muscle and that if not controlled, neutrophils cause further damage at the injury site within the muscle.

Muscle repair is a complex process that includes the invasion of neutrophils which dominate inflammation and early injury response whereas repair and muscle remodeling is primarily handled by the macrophages already present or recruited to the injury site (Butterfield et al., 2006). After an injury, there are two non-resident macrophages (M1 and M2) as well as two resident macrophages, ED1^+^ and ED2^+^, present at the injury site (Butterfield et al., 2006). ED1^+^ macrophages are present within necrotic muscle tissue as soon as within 1 day of injury and are activated by pro-inflammatory cytokines like TNF-α and IL-1β (Hirani et al., 2001). To magnify the immune response, these macrophages, as well as M1 macrophages, release over 100 molecules including cytokines to recruit more macrophages and neutrophils (Scott et al., 2004). Within the first few days post-injury, they work along with neutrophils to remove debris and dead tissue from the injured muscle site (Butterfield et al., 2006).

Unlike ED1^+^ macrophages, ED2^+^ and M2 macrophages are not found within necrotic tissue and play a primary role in repair and remodeling (Butterfield et al., 2006). ED2^+^ macrophages are found within the ECM of injured muscles and are responsible for cell signaling and cytokine production to induce muscle repair and regeneration (St Pierre and Tidball, 1985). In repair, the most important growth factors released by macrophages are fibroblast growth factor (FGF), insulin-like growth factor (IGF-1), and transforming growth factor–β1 (TGF-β1) (Butterfield et al., 2006). These cytokines recruit and activate fibroblasts that release matrix-building molecules such as collagen to begin the muscular repair process (Butterfield et al., 2006). As this tissue repair process is underway, fibroblasts continue releasing pro-inflammatory cytokines like IL-6 and IL-1 recruiting additional fibroblasts and neutrophils to the injury site (Cannon and St Pierre, 1998). Fibroblasts generate new ECM and deposit collagen supporting the healing and regenerating tissue (Bainbridge, 2013). While active, fibroblasts constantly secrete other ECM molecules such as proteoglycans, fibronectin, tenascin, laminin, and fibronectin creating a strong foundation for the repair and regeneration of muscle fibers via satellite cells (McAnulty, 2007).

Satellite cells in the muscle are active members of repair as they fuse to repair and form new muscle fibers at the injury site (Rathbone et al., 2003). In healthy muscular tissue, satellite cells are inactive and express only Pax7, but not Myod or Myogenin (Yin et al., 2013). When exposed to damaged tissue, satellite cells across the entire muscle fiber are activated, proliferate, and migrate to the injury site (Schultz et al., 1985). Once active and at the injury site, satellite cells differentiate into myogenic precursor cells or myoblasts that express myogenic transcription factors Myod and Myogenin (Yin et al., 2013). This activation, differentiation, and expression can be observed as early as 12 h after injury giving insight into the complexity of the inflammatory and repair response (Rantanen et al., 1995). These myoblasts fuse to create nascent myotubes containing few nuclei with cell membrane proteins β1-integrin, integrin receptor V-CAM, VLA-4 integrin, transcription factor FKHR, and caveolin-3 being key molecules (Yin et al., 2013). Over time, more myoblasts fuse, increasing the size of the myotube forming a mature muscle fiber capable of contracting (Yin et al., 2013).

Although both are necessary for wound healing, fibroblasts and muscle satellite cells must be properly balanced to ensure effective regeneration. While both cell types are essential, the over-activation of fibroblasts presents a significant barrier to recovery. This excessive fibroblast activity leads to fibrosis—the deposition of dense, disorganized extracellular matrix (ECM) or scar tissue—which physically impedes muscle regeneration and ultimately results in persistent loss of contractile function (Serrano et al., 2011). Current clinical mainstays like movement and stretching are crucial for mitigating this fibrotic response (Gardner et al., 2020), but there’s a clear need for more potent therapeutic interventions.

This critical balance between muscle regeneration and detrimental fibrosis can also be influenced by hormonal differences between men and women. Testosterone generally accelerates healing in men by boosting protein synthesis, which can result in faster, more robust muscle recovery and growth post-injury (Herbst and Bhasin, 2004). Estrogen presents a more complex picture: while it may initially protect muscle cells from damage, its effect on the speed of the repair process itself, especially when compared to testosterone, is uncertain and likely context-dependent (Chidi-Ogbolu and Baar, 2018).

### Endogenous repair of tendon injuries

Similar to muscle injury, tendon injuries can occur in many ways leading to pain and a decrease in function. The two most common injuries of tendinous structures are ruptures and overuse injuries (tendinopathy) (Sharma and Maffulli, 2005; Thomopoulos et al., 2015). Ruptures or tears occur when the tendon is acutely overloaded or lacerated while tendinopathy occurs over time due to excessive use or age-related degeneration (Xu and Murrell, 2008). In rupture or laceration injuries, the tendon’s structure and distribution of collagen is heavily disrupted and often require surgical intervention to realign and secure the injured tendon (Thomopoulos et al., 2015; Järvinen et al., 2004). Differing from muscle, the healing potential of a tendon that has been injured is heavily dependent upon the location of the tendon and whether or not it is encompassed by a synovial sheath (Thomopoulos et al., 2015).

Intrasynovial tendons, which are tendons that are surrounded by a synovial sheath, are lubricated by synovial fluid which is vital for decreasing friction and limiting wear from constant movement (Themes, 2016). Some examples of these tendons are in sections of the body that require the most movement such as the dorsal wrist area, the posterior tibial tendon at the medial malleolus, and the biceps brachii tendons (Themes, 2016). Extrasynovial tendons, which are tendons that are not encompassed by a synovial sheath, are found in the subcutaneous soft tissue (Themes, 2016). Not required for consistent movement, these tendons do not contain the lubrication of synovial fluid and therefore respond very differently to injury (Shen et al., 2021).

Although healing potential is dependent upon a few different factors, the general healing process of an injured tendon is similar to that of muscle as it follows an inflammation phase, a proliferative phase, and a remodeling phase (beginning post-procedure if surgical intervention is required) (Voleti et al., 2012). During the first stage of healing, the inflammatory phase, tendons become more vascularly permeable as an influx of inflammatory cells infiltrates (Thomopoulos et al., 2015). However, the degree of vasculature varies. A study comprising adult canines observed the difference in healing following a flexor tendon transection that was surgically repaired in both intrasynovial and extrasynovial tendons (Shen et al., 2021). Distal flexor tendons, which are intrasynovial, remained largely avascular 7 days post-injury leading to a silenced inflammatory response resulting in poor clinical outcomes (decrease in range of motion of digits) (Shen et al., 2021). In contrast, proximal flexor tendon injuries, which are extrasynovial, demonstrated dense vascularization leading to an increased cellular response and better healing 7 days post-injury (Shen et al., 2021).

Similar to muscle, the inflammation response in tendons leads to the recruitment and activation of inflammatory cells such as neutrophils, macrophages, and eventually fibroblasts (Sharma and Maffulli, 2005). In some cases where injuries require tendon-to-bone repair, osteoclasts are recruited to the injury site as well (Thomopoulos et al., 2015). As stated previously, the efficiency these cells are delivered to the injury site is largely dependent upon their surrounding environment and the vascularity present (Marsolais et al., 2001). M1 and M2 macrophages continue recruiting and clearing debris transitioning the injury site from inflammation/proliferation to repair and remodeling. Growth factors like TGF-β, vascular endothelial growth factor (VEGF), platelet-derived growth factor (PDGF), and FGF are released and are largely responsible for the beginning sections of the healing process in tendons (Molloy et al., 2003). Once again like muscle, the healing of tendons is heavily dependent upon the balance of pro and anti-inflammatory cells.

**FIGURE 4:**
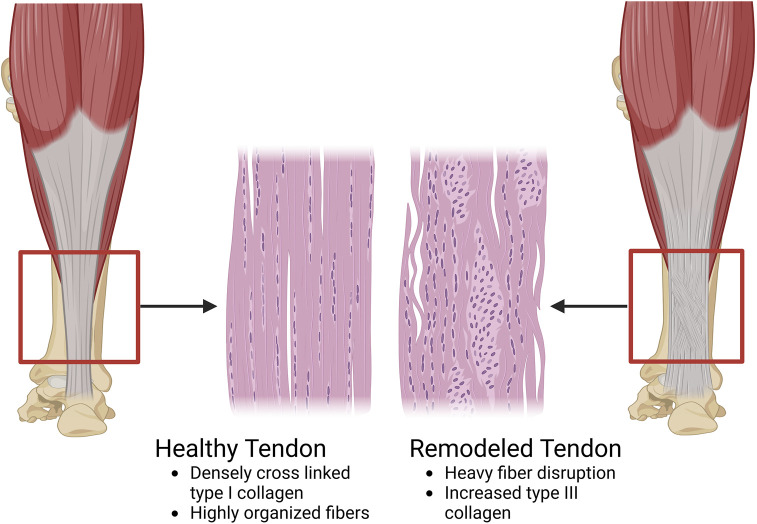
A diagram of the compositional and structural differences between a healthy tendon and a tendon undergoing remodeling. In the healthy tendon, the composition is predominantly type I collagen (65%–80% of the dry mass of the tendon). These collagen molecules are organized in a highly anisotropic and hierarchical manner with alignment of each collagen fibril and fiber to the long axis of the tendon. Additionally, the healthy tendon is hypocellular. On the other hand, in the remodeled tendon, type III collagen is present in a higher concentration than normal. Type III collagen is smaller and easier to manufacture than type I collagen, however it is also less organized which leads to a loss in the anisotropic structure of the healthy tendon. Along with the increased type III collagen, there is a greater infiltration of cells, both tenocytes, and other populations like macrophages (Image created at https://BioRender.com).

Once fibroblasts are recruited to the injury site via macrophages, they begin depositing collagen (Sharma and Maffulli, 2005). Unlike the heavily cross-linked, tightly woven type I collagen of healthy tendons, fibroblasts randomly deposit primarily type III collagen to begin remodeling the tendon (Liu et al., 1995; Nichols et al., 2019). Due to the lack of organization and differing collagen (Figure 4), the healed tendon has suboptimal performance levels compared to a non-injured tendon (Sharma and Maffulli, 2005; Järvinen et al., 2004; Obst et al., 2018). Furthermore, although tendons go through a rigorous healing phase, full recovery is incredibly difficult. Depending on the location, tendons have a retear rate of up to 35% and 94% post-surgery (Andarawis-Puri et al., 2015). This depends upon many factors such as age, tendon tear size, systemic diseases, and fatty infiltration (Andarawis-Puri et al., 2015). In the cases of overuse injuries such as tendinopathy, significant degeneration can take place leading to similar results with decreased functionality (Obst et al., 2018; Arya and Kulig, 2010).

Adding another layer of complexity to this healing process, tendon repair outcomes are also significantly influenced by sex-specific hormonal differences. In women, estrogen influences healing via receptors on tendons and ligaments, affecting collagen production, tissue remodeling enzymes (MMPs), and inflammation (Chidi-Ogbolu and Baar, 2018; Tanaka et al., 2019; Tashjian et al., 2021; Knewtson et al., 2022). Research suggests this might lead to slower collagen linking and less stiff scar tissue, potentially impacting tendon strength, though clinical significance is still under investigation. Conversely, testosterone in men also affects collagen synthesis and may promote faster, stronger tendon healing, although this requires more study. Additionally, sex hormones modulate the immune response, leading to different inflammatory cell activity (like macrophages) between sexes, which can alter the healing timeline and outcome.

### Tissue crosstalk during endogenous repair

When injuries take place at or near the MTJ, both tendon and muscle cells within the MTJ release signals to ensure proper repair can take place (Shama et al., 2024). A healthy MTJ structure is primarily composed of multinucleated myofibers and tenocytes (Shama et al., 2024). There are also immune cells (resident macrophages), stromal cells, endothelial cells, nerve cells, tendon fibroblasts, and progenitor cells which are all essential for upkeep and repair after injury to the MTJ (Ross et al., 2023; Bakooshli et al., 2018; Kostrominova et al., 2009). Upon injury to the MTJ, these cells work together to initiate the healing process. Cells within the muscular portion of the MTJ release TGF-β and IGF-1 while the tendinous portion of the MTJ releases IGF-1 and many cytokines in response to injury (Shama et al., 2024). The bioavailability of these signaling molecules is heavily regulated by binding to the MTJ’s ECM and proteoglycans (Cramer and Badylak, 2020). These soluble factors increase the interaction between the muscle and the tendon at the MTJ as the injury progresses through the stages of healing (Curzi et al., 2016). Although the exact chemical interactions at the MTJ are still relatively unknown during healing, a strong belief is that it repairs just as muscles or tendons do independently, except for it being heavily regulated by the ECM and proteoglycans present at the intersection of the two tissues (Snow et al., 2024).

### Putative therapies for accelerating endogenous healing

The pursuit of effective treatments for muscle, tendon, and MTJ injuries has spurred investigations into a wide array of therapeutic options. While some, like anabolic agents (e.g., IGF1) and metabolic reprogramming drugs (e.g., pioglitazone) are enticing due to their natural presence of their targets at the MTJ (Shama et al., 2024; Ladd et al., 2011) and their ability to enhance endogenous healing processes (Clark et al., 2023; Liang and Ward, 2006; Southern et al., 2019; Disser et al., 2019), their translational potential is hindered by the need for further targeted research and robust clinical data that mirrors their preclinical success.

Beyond growth factors or cytokines, scaffold therapies, aiming to provide both mechanical support and a healing environment, have garnered significant interest. Electrospun aligned scaffolds, with and without a cellular component, have received a lot of interest in recent years for its ability to form scaffolds with similar fiber structures and alignment to native tendons (Shama et al., 2024). Through creating gradients of different materials such as PCL and collagen Ladd et al. has even attempted to replicate the changes in mechanical properties along the MTJ (Shama et al., 2024; Ladd et al., 2011). Decellularized scaffolds have also received attention for their ability to preserve the tissues ultrastructure and ECM composition (Shama et al., 2024). While these structural therapies have promise, there remains limitations particular around balancing bioactivity and mechanical properties, as well as sterility and anchoring methods that will not degrade to weaken either the construct or the tissue further.

Cell-based therapies provide another tool that is seen as a viable option for promoting repair and remodeling. For tendons, Schnabel et al. and Koch et al. have shown mesenchymal stem cells (MSCs) in conjunction with IGF1 or through activation via exposure to inflammatory cytokines promote tendon healing and remodeling within an equine tendinopathy model (Schnabel et al., 2009; Koch and Schnabel, 2023). MSCs are an ideal cell therapy as they have the capacity to differentiate into multiple MSK cell types and can be collected autologously (Tsai et al., 2021). Work is ongoing for the translation of MSCs to human application, injections of MSCs are currently being used, though not FDA approved.

Physical therapy is a cornerstone of injury rehabilitation, focusing on the mechanosensitive properties of muscle and tendon to induce a cellular response for remodeling and repair (Killian et al., 2012; Ng et al., 2017; Thompson et al., 2016). For physical therapy, it is important to balance rest and reduction of load with loading of the tissues to promote healing, but not lead to either atrophy from lack of use or further damage from overuse (Killian et al., 2012). Physical therapy, however, it is most effective when integrated with other therapeutic modalities. While physical therapy is a promising approach for stimulating endogenous healing and rehabilitation, it is unlikely to lead to full recovery from injury on its own. Optimal treatment regimens should combine physical therapy with other promising approaches that stimulate the body’s natural healing processes.

While the research landscape presents an expanding portfolio of potential therapies for musculoskeletal injuries, the translation of these promising concepts from the laboratory bench to widespread clinical application confronts a significant gap. This translational inertia stems from multiple factors, including the intricate biology of tissue repair, the substantial financial investment and time required for rigorous clinical trials, and the challenges of developing preclinical models that accurately reflect human conditions. Therefore, advancing treatment likely necessitates moving beyond single modalities towards more sophisticated, multi-faceted approaches.

However, realizing this vision requires overcoming considerable real-world obstacles. Navigating the regulatory environment presents a major hurdle, particularly for novel biologics, cell therapies, and especially combination products, which often face more complex approval pathways than traditional pharmaceuticals or established procedures like physical therapy. Concurrently, ensuring patient access and equitable delivery involves tackling significant economic challenges. The potentially high cost of innovative therapies must be reconciled with healthcare system budgets and reimbursement policies, which increasingly demand compelling evidence of cost-effectiveness alongside clinical efficacy.

Ultimately, the successful clinical integration and justification for any therapeutic strategy, especially novel or combined approaches, critically depend on the generation of high-quality, compelling clinical evidence. Robust data from well-designed trials, demonstrating not only statistically significant improvements but also clinically meaningful and durable long-term functional outcomes, patient-reported benefits, and a favorable safety profile, is indispensable. Such evidence is the cornerstone for obtaining regulatory approval, securing reimbursement from payers, guiding clinical practice guidelines, and truly elevating the standard of care for individuals suffering from musculotendinous injuries.

## Conclusion

MSKIs represent a primary cause of disability among military personnel. Within this category, overuse injuries are the most prevalent, and musculotendinous overuse injuries prove particularly challenging due to their typically long recovery periods. This situation highlights the limitations of current therapeutic options for accelerating healing, underscoring the urgent need for a better understanding of the underlying injury and repair mechanisms to develop more effective treatments. Consequently, promising research directions are emerging, including therapies aimed at supporting endogenous healing processes—such as anabolic agents or metabolic reprogramming drugs—as well as scaffold or cell-based approaches and the optimization of physical therapy protocols. However, while much research focuses on restoring the structural components crucial for musculotendinous function, achieving optimal functional recovery also necessitates considering neurophysiological factors, which warrant further exploration. Given the complexity of these injuries and the potential benefits of new approaches, further investment of research funding and effort targeting musculotendinous injury therapies is clearly justified. For the military, in particular, such investment holds significant potential, as effective treatments would directly translate into reduced lost duty days, decreased medical costs, and ultimately, improved overall readiness.
